# Study on the Single Sheet Measurement Method for AC Magnetic Measurement on Grain-Oriented Electrical Steel

**DOI:** 10.3390/ma16041648

**Published:** 2023-02-16

**Authors:** Qian Xiang, Lin Cheng, Kaiming Wu

**Affiliations:** 1 The State Key Laboratory of Refractories and Metallurgy, Hubei Province Key Laboratory of Systems Science on Metallurgical Processing, International Research Institute for Steel Technology, Collaborative Center on Advanced Steels, Wuhan University of Science and Technology, Wuhan 430081, China; 2 Wuhan Iron and Steel Co., Ltd., Wuhan 430081, China

**Keywords:** grain-oriented electrical steel, H-coil method, single sheet tester (SST), effective magnetic path length, magnetizing current (MC) method

## Abstract

To quickly and accurately measure the AC magnetic properties of grain-oriented electrical steel by means of the existing measuring system designed for the magnetizing current method (MC), specifically the SST (92) single sheet method, in this work, the H-coil (HC) measuring system, which directly senses the magnetic field strength of the tested sample, was designed to measure the AC magnetic properties of the grain-oriented electrical steel. The assumed effective magnetic path length introduced in the MC method was corrected by comparing the measurement results obtained by means of HC and MC methods. The results found that specific total loss measured by the HC method was significantly lower than that measured by the classical magnetizing current (MC) method. Taking the HC method as the reference, the influencing factors of the effective magnetic path length was studied. It was found that the actual effective magnetic path length depends on the investigated sample characteristics, the measurement conditions, as well as yoke characteristics. The actual effective magnetic path length introduced in the MC method is examined to be more than 450 mm, fluctuating around 468 mm.

## 1. Introduction

Cold-rolled, grain-oriented electrical steel produced by the cold-rolling process exhibits excellent magnetic properties due to the (110) <001> Gauss texture in the rolling direction and has been widely utilized as an iron core material of the electrical products serviced under directional magnetic field, such as transformers, ballasts, amplifiers, regulators, relays, rectifiers, electromagnetic switches, etc. [1,2]. In industrial applications, the quality of grain-oriented electrical steel is mainly evaluated by two AC magnetic properties indexes, i.e., the specific total loss and magnetic polarization strength. At present, the internationally recognized and commonly used methods for measuring AC magnetic properties are the Epstein frame method and the single sheet test method (SST). So far, the measurement of AC magnetic properties of grain-oriented electrical steel is still an international problem [3,4]. For the Epstein frame method, samples with sizes of 300 mm × 30 mm are divided into four groups, which are lapped with the neighbors to form the closed magnetic circuit. A no-load transformer is then formed by the lapped samples and the primary and secondary windings winded on the samples. The assumed effective magnetic path for the Epstein frame method is 0.94 m. The SST measuring system [5,6,7] consists of two separated C-type magnetic yokes. A closed magnetic circuit is formed by joining the upper and lower yokes with the measured sample (500 × 500 mm). There are two windings winded around the samples between the upper and lower yokes. The outer one is the primary winding (magnetizing winding), and the inner one is the secondary winding (induced voltage winding). Due to the sample of smaller size required by the Epstein frame method, stress-relief annealing must be conducted on the processed samples, and several pieces of samples are needed in one turn of measurement, which are the main disadvantages of the Epstein frame method. Although only one sample with larger size is required for the SST method, the effective magnetic path length introduced in the SST method should be corrected.

In the current IEC 60404-3:2010 “Magnetic materials-Part 3: Methods of measurement of the magnetic properties of electrical steel strip and sheet by means of a single sheet tester” [7], there are two definitions of the effective magnetic path length [3]. Firstly, for the sake of measurement consistency, the effective magnetic path length is considered as the inner length of the yoke (450 mm), which is referred to as the SST (92) method. Secondly, the effective magnetic path length of the single yoke is traced based on the measurement results using the Epstein frame method, which is called the SST (82) method for short. The SST (82) method cannot achieve measurement consistency between domestic and foreign laboratories due to the measurement fluctuation in the Epstein frame method. Systematic deviation exists between these two methods demonstrated due to the different definition of effective magnetic path length. Statistics of measurement results demonstrate that the specific total loss measured by the SST (92) method is at least 5% higher than that measured by the SST (82) method because the effective magnetic path length of 450 mm utilized in the SST (92) method does not include the part where the sample contacts the magnetic pole surface of the yokes [8]. However, in order to ensure the consistency between the laboratories, the SST (92) method is internationally adopted by the major laboratories, which indicates that, for the same sample, the specific total loss measured by the SST (92) method is about 5% higher than that measured by the Epstein frame method. On the other hand, the magnetic properties of the grain-oriented electrical steels with refined magnetic domain can only be measured by means of the SST (92) method because the stress-relief annealing required by the Epstein frame method could also eliminate the magnetic domain refinement effect; meanwhile, since the measured specific total loss is closer to the true value, the Epstein frame method is generally adopted for industrial application at home and abroad. Therefore, within the electrical steel industry, it has been always a goal to try to effectively eliminate the systematic deviation of the single sheet measurement method at home and abroad.

Up to now, clearer or more effective methods of defining the effective magnetic path length required in the SST (92) method have not been provided [9,10]. Therefore, the 2020 grain-oriented silicon steel standard (IEC 60404-8-7) proposed a temporary solution by introducing the conversion factor to correct the measurement obtained by means of the SST (92) method [11]. Specifically, by multiplying by a conversion factor of 0.925, the specific total loss measured at 1.7 T and 50 Hz (60 Hz) by the SST method can be treated as equal to the one measured by the Epstein frame method. However, this solution has not reached a broad consensus in the electrical steel production and application industry because it did not fundamentally eliminate the systematic deviation existing between these measurement methods.

In order to fundamentally solve the effective magnetic path length problem in the SST (92) method, Japanese experts put forward the HC method in the 1960s and 1970s [12,13,14,15]. The main difference between the HC method and the MC method is that the HC method no longer needs the assumed effective magnetic circuit length, which is a fundamental change in the measuring principle. Since the sample thickness is less than 1/100 of the yoke width, the voltage signal detected by the H-coil is only at millivolt-level, which is difficult to be measured and controlled accurately. Therefore, the HC method is not promoted globally. With the rapid development of manufacturing technology and measurement science in the past ten years, the measurement of AC magnetic properties of grain-oriented electrical steel by mean of the H-coil method has gradually become a reality.

At present, the SST (92) single sheet (MC) method is widely utilized in the electrical steel industry at home and abroad. However, the testing equipment designed for the MC method does not have the H-coil measurement function. In addition, few laboratories have the ability to develop their own HC measuring system. Therefore, how to quickly and accurately measure the AC magnetic properties of the grain-oriented electrical steel under the existing measuring system has become a hot topic in the electrical steel industry. In this work, based on the newly designed measuring system integrating HC and MC methods, the systematic deviation between MC and HC methods in measuring the magnetic properties of grain-oriented electrical steel was investigated. The methods to solve the accuracy of AC magnetic property measurement are given. New insights have been given to solve the accuracy problems in AC magnetic properties measurement under current measuring conditions in the electrical steel industry.

## 2. Methodology

### 2.1. Measuring Principle of the Magnetizing Current Method (MC)

MC is an indirect measurement method and has obvious defects due to the introduction of the effective magnetic path length hypothesis. Its main idea is to calculate the magnetic field strength (*H*) based on the magnetizing current [6], as shown in Equation (1).

(1) $$H=\frac{N_{1}}{l_{m}}I$$

where *N*_1_ is the number of turns in the primary coil of the permeameter and *l_m_* is the effective magnetic path length. *l_m_* is assumed to be equal to the inner length of the yoke, based on the hypothesis that “the magnetic field strength in the remained closed magnetic circuits is zero”. However, this simplified hypothesis cannot be held because the effective magnetic path length of 450 mm utilized in the SST (92) method does not include the part where the sample contacts the magnetic pole surface of the yokes.

### 2.2. System Structure and Measuring Principle of H-coil

**Figure 1:**
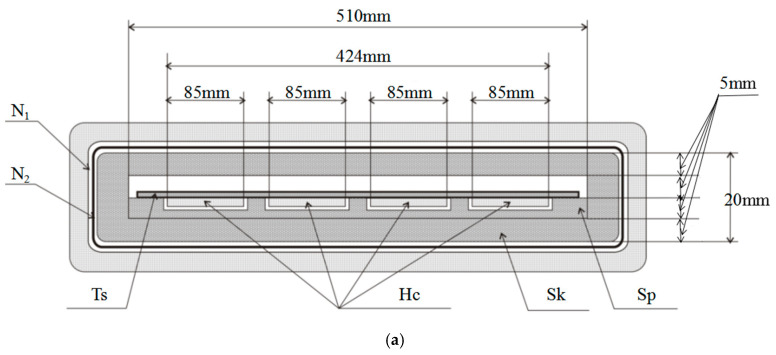

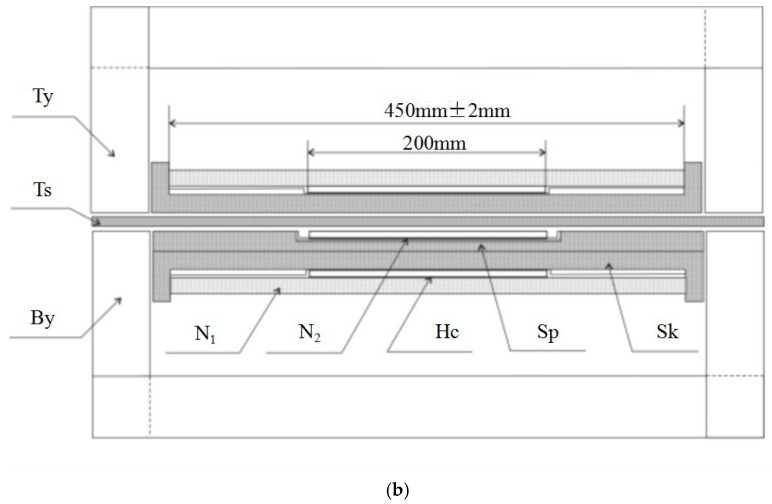
Schematic diagram of magnetic yoke winding. (**a**) Cross-section diagram. (**b**) Vertical section diagram. *N*_1_: The primary winding; *N*_2_: the secondary winding; Ts: sample; H: H-coil; Sk: skeleton; Sp: mounting plate; Ty: upper yoke; and By: lower yoke.

For the H-coil method, the sample is placed within the primary coil (*N_1_*, excitation coil) and the secondary coil (N_2_, B coil), as shown in Figure 1. The H-coil is placed near the lower surface of the sample to directly detect the magnetic field strength of the sample surface. The H-coil is composed of four coils in series. Each coil (200 ± 0.2 mm in length) is made with single layer copper wire with diameter of 0.2 mm continuously, evenly, and tightly wound onto a non-magnetic material at a size of 250 ± 1 mm × 85 ± 0.2 mm × 1 ± 0.1 mm.

**Figure 2:**
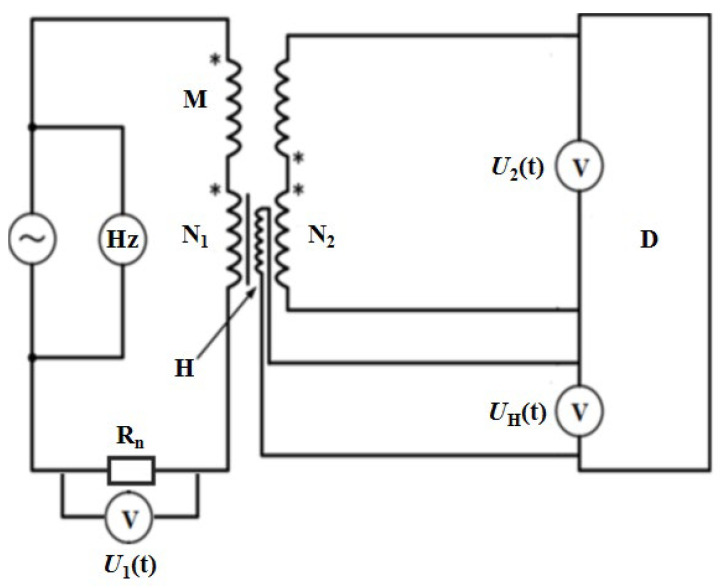
Schematic diagram of the measurement principle of the H-coil method. ~: power supply; Hz: frequency meter; *N*_1_: the primary winding; *N*_2_: the secondary winding; *: homonymous ends; H: H-coil; R_n_: non-inductive precision resistor; D: digitizer; *U*_2_(*t*): voltage induced in the secondary winding; *U_H_*(*t*): voltage induced in the H-coil; *U*_1_(*t*): voltage drop across the non-inductive precision resistor; and M: air flux compensation coil, which can be removed when using digital flux compensation.

The sample and the U-shaped upper and lower magnetic yokes form a closed magnetic field circuit. The cross-sectional area of the yoke is much larger than the cross-sectional area of the sample. As shown in Figure 1, magnetic field strength near the sample surface can be measured by detecting the induced potential within the H-coil. Magnetic flux density can be examined by measuring the induced potential on the secondary coil. The circuit diagram of the measurement process is shown in Figure 2. The waveform, the voltage *U_1_*(*t*) related to the primary winding, *U_2_*(*t*) of the secondary winding, and the *U_H_* (*t*) of the H-coil are recorded in real time through the digital control system.

Magnetic field strength *H*(*t*) and magnetic polarization intensity *J*(*t*) at time *t* can be calculated by Equations (2) and (3) [12], respectively.

(2) $$H\left(t\right)=\frac{1}{\mu_{0}\left(N_{H}A_{H}\right)}\left\{\overset{t}{\underset{0}{\int }}U_{H}\left(\tau \right)d\tau -\frac{1}{T}\int_{0}^{T}\left(\int_{0}^{t}U_{H}\left(\tau \right)d\tau \right)dt\right\}$$

(3) $$J\left(t\right)=\frac{1}{N_{2}A}\left\{\overset{t}{\underset{0}{\int }}U_{2}\left(\tau \right)d\tau -\frac{1}{T}\int_{0}^{T}\left(\int_{0}^{t}U_{2}\left(\tau \right)d\tau \right)dt\right\}$$

where *H*(*t*) is the magnetic field strength at time *t*, A/m; *µ*_0_ is the permeability of free space, H/m; *N_H_A_H_* is the total area of the H-coil, *m*^2^; *U_H_*(*t*) is the induced voltage in the H-coil at time *t*, *V*; *J*(*t*) is magnetic polarization intensity at time *t*, *T*; *N*_2_ is the number of the secondary coil; *U*_2_(*t*) is the induced voltage in the secondary coil at time *t*, *V*; and *A* is the cross-section area of the sample, *m*^2^.

The specific total loss is equivalent to the area of the hysteresis loop, which can be calculate by Equation (4) [12].

(4) $$P_{s}=\frac{f}{\rho_{m}}\int_{0}^{T}H(t)\frac{dJ(t)}{dt}dt=\frac{f}{\rho_{m}N_{2}A}\int_{0}^{T}H(t)U_{2}{}^{\prime }(t)dt$$

where *P_s_* is the specific total loss of the investigated sample, W/kg; *f* is the magnetizing frequency, *Hz*; *T* is equal to 1/f, *s*; and *U*_2_(*t*) is the secondary induced voltage with air flux compensation, *V*.

Specific apparent power, *S_s_*, is the product of the effective magnetic field strength and the effective magnetic polarization intensity, which can be calculated by Equation (5) [12].

(5) $$S_{S}=\frac{2\pi f}{\rho_{m}}\tilde{H}\tilde{J}$$

where *S_s_* is the specific apparent power, VA/kg; *H*-bar is the effective magnetic field strength, *A*/*m*; and *J*-bar is the effective magnetic polarization intensity, *T*.

### 2.3. Experimental Method

The authors, cooperating with TUNKIA Co., Ltd., have designed a new AC magnetic measurement equipment (TD8590), which integrates the HC and the MC (SST (92)) methods. The permeameter was made by laminating M100-27P5 grain-oriented electrical steel (without magnetic domain refinement) sheets to minimize the measurement error. This equipment was utilized in this work to conduct the magnetic properties measurement. For the experiment, 0.23 (M90-23P5, M95-23P5, M100-23P5)/0.27 (M95-27P5, M100-27P5, M110-27P5) mm grade grain-oriented electrical steels with and without the magnetic domain refinement were chosen because they are of the highest production. All the steels were supplied by China Baowu Group Corporation Limited. Si content varied in the range of 3.0–3.3 wt%. The magnetic properties of the grain-oriented electrical steel samples with the thickness of 0.23 mm and 0.27 mm without magnetic domain refinement treatment were measured in the comparative experiments. The total specific loss (P_1.7/50_) of these samples varied between 0.9 and 1.3 w/kg. With the magnetic polarization intensity varying in the range of 1.0 to 1.8 T, the AC magnetic properties at the frequency of 50 Hz and 60 Hz were measured at 0.1 T magnetic polarization intervals. The magnetic field strength, *H_m_*, coercive force, *H_c_*, residual magnetism, *Br*, permeability, μm, the specific total loss, *P_s_*, and the specific apparent power, *S_s_*, are measured. Then, the same process was conducted on the 0.23 mm and 0.27 mm grain-oriented electrical steel samples with magnetic domain refinement treatment. Taking magnetic properties measured by means of the HC method as the reference, the relative change of the counterpart measured by the MC method was calculated. Taking the specific total loss as an example, Δ*P_s_* can be calculated by Equation (6).

(6) $$\Delta P_{s}=\frac{P_{s}{}_{\left(HC\right)}-P_{s}{}_{\left(MC\right)}}{P_{s}{}_{\left(HC\right)}}\times 100\%$$

## 3. Experimental Results and Discussion

### 3.1. The Measurement Differences between the HC and MC Methods

Table 1 shows the relative difference between the magnetic properties measured by MC and HC methods. At the magnetizing frequency of 50 Hz, it can be seen that, to achieve the same magnetic polarization intensity, the magnetic field intensity, *H_m_*, needed for the HC method was lower than that needed for the MC method, which indicates that the permeability measured in the HC method was higher than that measured by the MC method. As the magnetic polarization increased from 1.0 T to 1.8 T, Δ*H_m_* gradually increased from −15.93% at 1.0 T to about −2.66% at 1.8 T, Δμm decreased from 13.63% to 2.29%, and Δ*H_c_* varied in the range of −9.0~−9.9%. Residual magnetism measured by the HC method was higher than that measured by the MC method. Δ*Br* decreased from 4.36% at 1.0 T to about 2.15% at 1.8 T. The specific total loss measured by the HC method was lower than that measured by the MC method. Δ*P_s_* increased from −8.69% at 1.0 T to about −6.57% at 1.8 T. The specific apparent power measured by the HC method was also lower than that measured by the MC method, and Δ*S_s_* gradually increased from −14.61% at 1.0 T to about −4.00% at 1.8 T. The variation law of these magnetic properties measured at the magnetizing frequency 60 Hz was not changed. 

**Figure 3:**
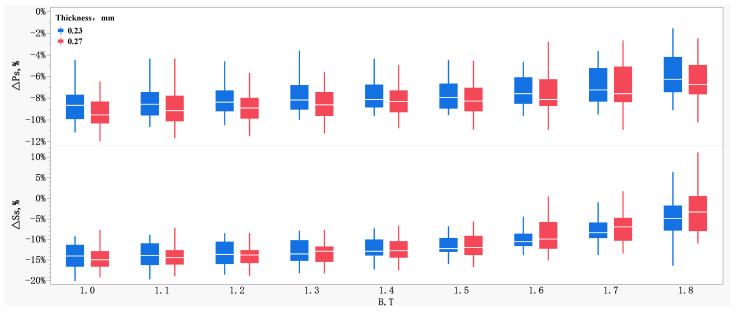
Effects of sample thickness on the relative variation of magnetic properties between the two measurement methods.

The effects of sample thickness on the relative change of magnetic properties measured by the two methods are demonstrated in Figure 3. It can be seen that the sample thickness had a significant impact on Δ*P_s_*. The thinner the sample, the smaller the Δ*P_s_*. As for the index of *P*_1.7/50_ or *P*_1.7/60_, the magnetic property measured by the HC method was lower than that measured by the MC method, by 7.2% on average, which is totally consistent with the conversion factor of 0.925 proposed in IEC 60404-8-7 [13]. At the same time, when the magnetic polarization intensity was lower than 1.6 T, the effects of the sample thickness on Δ*S_s_* are not significant, but when the magnetic polarization intensity was larger than 1.6 T, the Δ*S_s_* of the 0.23 mm sample was slightly greater than that of the 0.27 mm sample.

**Figure 4:**
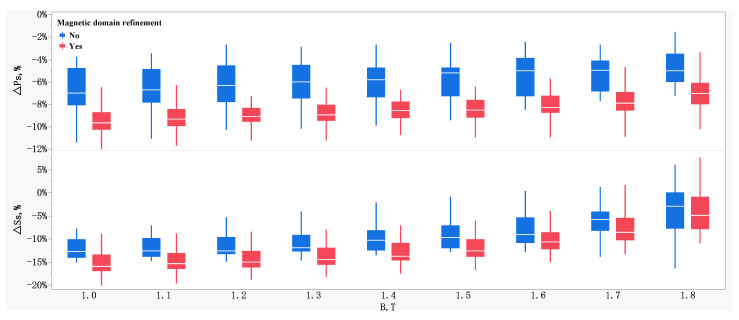
Effects of magnetic domain refinement on the relative deviation of magnetic properties measured by the two measurement methods.

**Figure 5:**
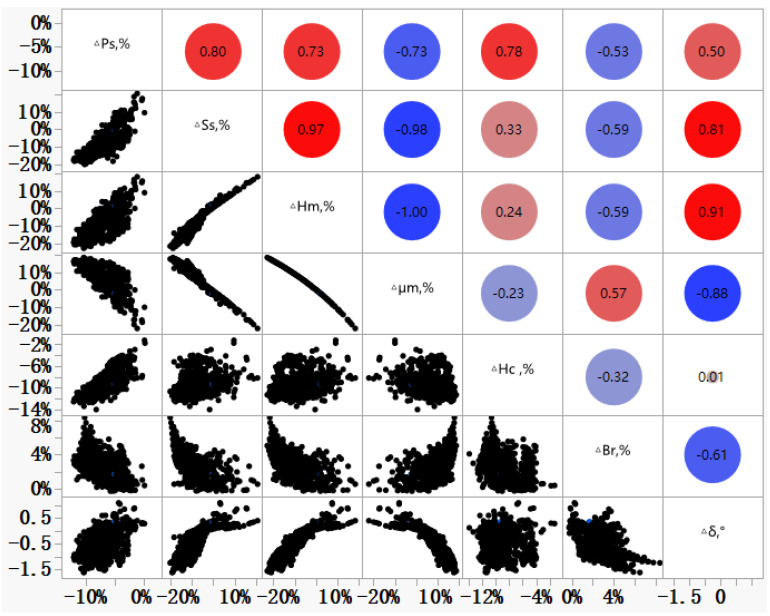
Correlation between the magnetic properties measured by the two measurement methods.

The effects of magnetic domain refinement on the relative change of magnetic properties measured by the two methods are shown in Figure 4. It can be seen that the relative change of Δ*P_s_* and Δ*S_s_* between the two measurement methods on the samples without magnetic domain refinement were smaller than those of the samples with magnetic domain refinement. Taking index *P*1.7/50 or *P*1.7/60 as an example, the Δ*P_s_* was about 5.1% for samples without magnetic domain refinement and about 7.5% for samples with magnetic domain refinement. Correspondingly, for *S*_1.7/50_ or *S*_1.7/60_, the Δ*S_s_* was about 6.0% for samples without magnetic domain refinement and about 7.6% for samples with magnetic domain refinement. Figure 5 exhibits the correlation diagram of the relative differences of physical properties measured by these two methods. It can be seen that the relative change of total specific loss, Δ*P_s_*, is highly related to Δ*H_m_*, Δ*H_c_*, and Δμm with the correlation coefficient more than 0.7. Meanwhile, the relative change rate of specific apparent power, Δ*S_s_*, is highly related to Δ*H_m_*, Δμm, and Δ*δ* with the correlation coefficient more than 0.8.

### 3.2. Measurement Deviation Caused by the Yokes

The above research results show that the index *P*_1.7/50_ or *P*_1.7/60_ of the electrical steel measured by the MC method was significantly higher than those measured by the HC method. This systematic deviation mainly comes from the effective magnetic path length assumption introduced in the MC method. The effective magnetic path length is closely related to the permeameters. Generally, the yoke in the permeameters is basically made of M100-27P5 grain-oriented electrical steel sheet without magnetic domain refinement by means of the *laminated object manufacturing method* (0.27 mm yoke for short). In this work, another permeameter was designed using M100-23P5 grain-oriented electrical steel sheet without magnetic domain refinement with the same processing techniques to study the influence of the permeameters on the total specific loss. The same batch of grain-oriented silicon steel sheets were chosen for the cross-test using these permeameters. Except for the permeameter, other magnetic measurement parameters were kept the same. The proportion of yoke loss within the total specific loss for different permeameters measured on 0.23 mm and 0.27 mm grain-oriented electrical steel sheets was examined by the MC method at the magnetizing frequencies of 50 Hz and 60 Hz [16]. Table 2 shows the experimental results. It was found that permeameters demonstrated obvious effects on the relative specific total loss. Under the common working magnetic polarization intensity range of 1.0–1.8 T for grain-oriented electrical steel, the ratio of specific total loss induced by the yoke (Δ*P_y−_*_0.23_) was decreased with the increasing magnetic polarization intensity, regardless of the types of permeameter and specimen thickness. For specific index *P*_1.7/50_ or *P*_1.7/60_, Δ*P_y−_*_0.27_ was larger than Δ*P_y-_*_0.23_ by about 1.0% for the sample with the thickness of 0.23 mm, and Δ*P_y−_*_0.27_ was larger than Δ*P_y−_*_0.23_ by about 0.65% for the sample with the thickness of 0.27 mm. Therefore, it can be concluded that the material and manufacturing process of the yoke also has a significant effect on the specific total loss.

### 3.3. The Correlation between HC and MC Methods

A large number of experiments have confirmed that the specific total loss, the specific apparent power, and other magnetic performance indexes of the grain-oriented electrical steel measured by the HC method are significantly lower than those measured by the MC method [15,16] because the HC method directly measures the magnetic field strength of the investigated sample without the involvement of the effective magnetic path length [15,17,18,19], and an assumed effective magnetic path length, not the actual effective magnetic path length, is suggested for the MC method. It is also found that the effective magnetic path length in the actual measurement is not a constant, which depends on the material and manufacturing process of the permeameters, the characteristics of the investigated sample, and the measurement conditions. In the literature [10,20,21,22], it is also found that the actual mean magnetic path length of the Epstein frame depends not only on the material permeability and anisotropy but also on the peak flux density and the magnetizing frequency.

**Figure 6:**
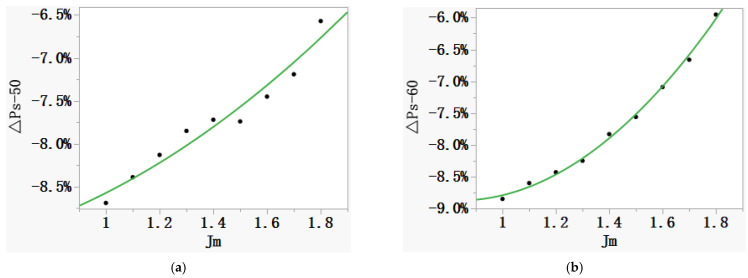
Quadratic relationship between *J_m_* and Δ*P_s_*. (**a**) 50 Hz. (**b**) 60 Hz.

**Figure 7:**
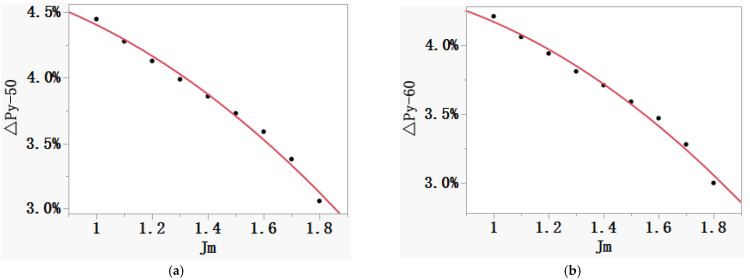
Quadratic relationship between *J_m_* and Δ*P_y_*. (**a**) 50 Hz. (**b**) 60 Hz.

However, the MC method is generally adopted for industrial application at home and abroad, and it is extremely difficult for laboratories to replace all the measuring systems at the same time, which makes it impossible to unify the measurement results through short-term equipment update. Therefore, dynamic adjustment on the effective magnetic path length in the MC method is an effective solution to reduce the deviation. First, based on the experimental data, the relationships between *J_m_* and Δ*P_s_* (see Table 1), and between *J_m_* and Δ*P_y_* (see Table 2), are established, as shown in Figure 6 and Figure 7. Then, the corrected effective magnetic path length can be obtained according to Equation (7).

(7) $$l_{m}^{\prime }=\frac{450}{1+\Delta P_{s}+\Delta P_{y}}$$

Taking the typical magnetic property index of grain-oriented electrical steel measured based on a common 0.27 mm yoke as an example, the corrected effective magnetic path length was examined based on the measured Δ*P_s_* and Δ*P_y_*, as shown in Table 3. It can be found that the actual effective magnetic path length was not a constant. It decreased slightly with increasing magnetic polarization intensity and fluctuated around 468 mm (significantly larger than the assumed 450 mm). It is worth noting that, if the systematic error caused by the yokes cannot be accurately examined, the effective magnetic path length used in the actual measurement could be set as 485 mm.

## 4. Conclusions

(1) The HC method eliminates the measurement systematic error caused by assumed effective magnetic path length, and the measured specific total loss, excitation power, and other magnetic performance indexes are significantly lower than those measured by the MC method. The HC method is a reliable alternative method for the magnetic property measurement on electrical steels in the future.
(2) The actual effective magnetic path length in the MC method can be examined based on the systematic deviation between the measurement results obtained by HC and MC methods under different magnetic polarization intensities with the specific total loss induced by the yoke deducted.
(3) The actual effective magnetic path length in the MC method is not a constant, which depends on the sample characteristics, the measurement conditions, and the material and manufacturing process of the permeameter. The actual effective magnetic path length of the MC method is larger than 450 mm, and fluctuates around 468 mm.
(4) Under the current measurement system of the single sheet method SST (92), the assumed effective magnetic circuit length of 468 mm is recommended; meanwhile, the final measurement result should be corrected according to the actual yoke loss.

## Figures and Tables

**Table 1:** Magnetic properties deviations between the two measurement methods.

*f*, Hz	*J_m_*, T	Δ*H_m_*, A/m	Δ*H_c_*, A/m	Δ*B_r_*, T	Δμm, mH/m	Δ*P_s_*, W/kg	Δ*S_s_*, VA/kg
50	1.0	−15.93%	−9.20%	4.36%	13.63%	−8.69%	−14.61%
	1.1	−16.05%	−9.13%	4.04%	13.77%	−8.39%	−14.18%
	1.2	−15.62%	−9.08%	3.62%	13.44%	−8.13%	−13.71%
	1.3	−14.53%	−9.05%	3.22%	12.62%	−7.85%	−13.03%
	1.4	−13.10%	−9.14%	2.82%	11.47%	−7.72%	−12.33%
	1.5	−11.43%	−9.45%	2.49%	10.10%	−7.74%	−11.46%
	1.6	−8.87%	−9.56%	2.21%	7.99%	−7.45%	−9.76%
	1.7	−5.68%	−9.86%	2.09%	5.21%	−7.19%	−7.38%
	1.8	−2.66%	−9.93%	2.15%	2.29%	−6.57%	−4.00%
60	1.0	−15.16%	−9.31%	3.76%	13.05%	−8.85%	−14.03%
	1.1	−15.43%	−9.23%	3.43%	13.26%	−8.60%	−13.69%
	1.2	−15.31%	−9.24%	3.05%	13.15%	−8.43%	−13.36%
	1.3	−14.44%	−9.29%	2.72%	12.48%	−8.25%	−12.87%
	1.4	−12.83%	−9.16%	2.42%	11.25%	−7.83%	−11.90%
	1.5	−11.11%	−9.19%	2.24%	9.87%	−7.56%	−10.97%
	1.6	−8.61%	−9.11%	2.09%	7.80%	−7.09%	−9.34%
	1.7	−5.37%	−9.09%	2.04%	4.96%	−6.66%	−6.97%
	1.8	−2.03%	−8.95%	2.09%	1.72%	−5.95%	−3.36%

**Table 2:** Relative change of specific total loss induced by the yokes.

Test Point, T	Sample Thickness, mm	The Yoke Loss Ratio of 0.23 mm Yoke Δ*P_y−_*_0.23_		The Yoke Loss Ratio of 0.27 mm Yoke Δ*P_y−_*_0.27_	
		50 Hz	60 Hz	50 Hz	60 Hz
1.0	0.23	2.94%	2.70%	3.90%	3.75%
1.1	0.23	2.79%	2.60%	3.80%	3.64%
1.2	0.23	2.66%	2.48%	3.69%	3.53%
1.3	0.23	2.54%	2.40%	3.57%	3.42%
1.4	0.23	2.43%	2.32%	3.45%	3.31%
1.5	0.23	2.31%	2.20%	3.33%	3.20%
1.6	0.23	2.20%	2.10%	3.18%	3.07%
1.7	0.23	2.04%	1.97%	2.97%	2.89%
1.8	0.23	1.74%	1.72%	2.58%	2.54%
1.0	0.27	3.83%	3.50%	4.45%	4.21%
1.1	0.27	3.67%	3.37%	4.28%	4.06%
1.2	0.27	3.46%	3.23%	4.13%	3.94%
1.3	0.27	3.28%	3.11%	3.99%	3.81%
1.4	0.27	3.18%	3.02%	3.86%	3.71%
1.5	0.27	3.05%	2.92%	3.73%	3.59%
1.6	0.27	2.91%	2.79%	3.59%	3.47%
1.7	0.27	2.79%	2.65%	3.38%	3.28%
1.8	0.27	2.48%	2.42%	3.06%	3.00%

**Table 3:** Corrected effective magnetic path length for 0.27 mm permeameters.

Test Point, T	Δ*P_s_*, %		Δ*P_y_*, %		$l_{m}^{\prime }$	
	50 Hz	60 Hz	50 Hz	60 Hz	50 Hz	60 Hz
1.0	−8.57%	−8.79%	4.41%	4.17%	469.57	471.80
1.1	−8.41%	−8.66%	4.29%	4.08%	469.30	471.61
1.2	−8.22%	−8.47%	4.17%	3.97%	469.01	471.18
1.3	−8.02%	−8.21%	4.03%	3.85%	468.71	470.52
1.4	−7.80%	−7.89%	3.88%	3.72%	468.40	469.62
1.5	−7.57%	−7.52%	3.71%	3.57%	468.07	468.48
1.6	−7.32%	−7.08%	3.53%	3.41%	467.73	467.12
1.7	−7.05%	−6.58%	3.33%	3.24%	467.37	465.54
1.8	−6.77%	−6.02%	3.13%	3.06%	467.00	463.73
Mean Value					468.35	468.84

## Data Availability

The data are available from the corresponding author on reasonable request.
